# UV-B induced transcript accumulation of DAHP synthase in suspension-cultured *Catharanthus roseus *cells

**DOI:** 10.1186/1750-2187-5-13

**Published:** 2010-08-13

**Authors:** Shilpa Ramani, Nandadevi Patil, Chelliah Jayabaskaran

**Affiliations:** 1 Department of Biochemistry, Indian Institute of Science, Bangalore-560012, India

## Abstract

The enzyme 3-deoxy-D-arabino-heptulosonate-7-phosphate (DAHP) synthase (EC 4.1.2.15) catalyzes the first committed step in the shikimate pathway of tryptophan synthesis, an important precursor for the production of terpenoid indole alkaloids (TIAs). A full-length cDNA encoding nuclear coded chloroplast-specific DAHP synthase transcript was isolated from a *Catharanthus roseus *cDNA library. This had high sequence similarity with other members of plant DAHP synthase family. This transcript accumulated in suspension cultured *C. roseus *cells on ultraviolet (UV-B) irradiation. Pretreatment of *C.roseus *cells with variety of agents such as suramin, N-acetyl cysteine, and inhibitors of calcium fluxes and protein kinases and MAP kinase prevented this effect of UV-B irriadiation. These data further show that the essential components of the signaling pathway involved in accumulation DAHP synthase transcript in C*. roseus *cells include suramin-sensitive cell surface receptor, staurosporine-sensitive protein kinase and MAP kinase.

## Background

The first enzyme of the shikimate pathway catalyzes the condensation of phosphoenolpyruvate and erythrose-4-phosphate to yield DAHP. DAHP synthase is reportedly induced by abiotic stresses such as mechanical wounding [1]. The first evidence of metabolic regulation of a plant DAHP synthase came from experiments with suspension cultured potato cells, exposed to glyphosate [2]. Metabolic regulation of DAHP synthase in plants appears to occur preferentially at the transcriptional level. DAHP synthase transcript was found to accumulate in response to several environmental stimuli that also induced phenylalanine ammonia lyase (PAL) mRNA [1,3]. This suggests that the synthesis of aromatic amino acids might be regulated in concert at the transcriptional level. Several isozymes specific to cytosolic and plastid have been reported for plant DAHP synthase [4]. The elicitor treatment of parsley cell suspensions or wounding of potato tubers induced DAHP synthase isoenzyme specific to plastid but not the putative cytosolic form [5,6]. Isolation and characterization of cDNA that encodes DAHP synthase from *Catharanthus roseus *and accumlation of its transcript and the signaling components involved have been described in this study.

## Results

### Isolation and characterization of cDNA of a DAHP synthase preferentially expressed in UV-B-treated C. roseus cultured cells

**Figure 1:**
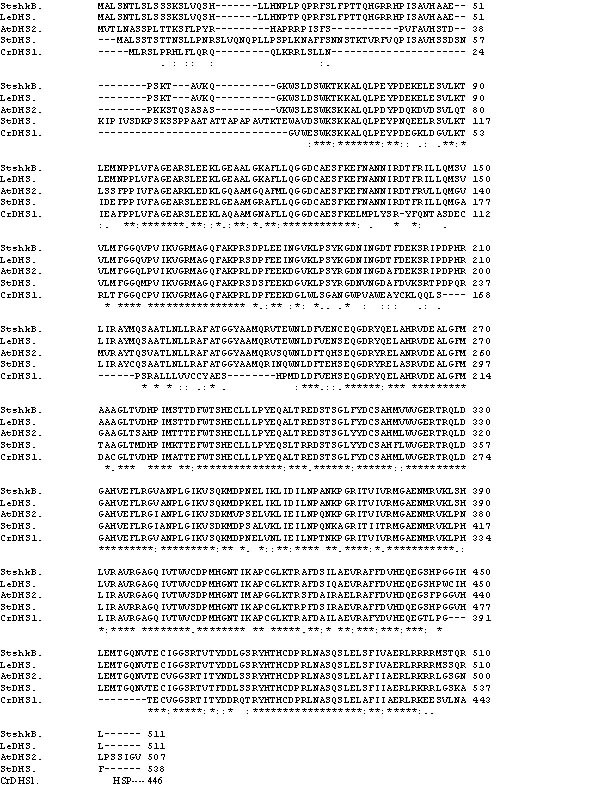
**Comparison of the deduced amino acid sequence of ***CrDHS1 ***with known plant nuclear coded chloroplast-specific DHS proteins**. Sequences were aligned using ClustalW program located at ExPasy site http://www.expasy.ch. Sequence descriptions and Genbank accession numbers are: StSHKB, *S. tuberosum *deoxy arabino heptulosonate phosphate synthase (M95201); LeDHS, *L. esculentum *deoxy arabino heptulosonate phosphate synthase (Z21792); AtDHS, *A. thaliana *deoxy arabino heptulosonate phosphate synthase (M74820); StDHS, *S. tuberosum *deoxy arabino heptulosonate phosphate synthase (AB061254), and *CrDHS1*, *C. roseus *deoxy arabino heptulosonate phosphate synthase. Numbers of amino acids are indicated on the right. Dashes indicate gaps introduced in order to optimize the alignment. Asterisks (*) indicate conserved residues in all DHS sequences. Single or double dots represent similar amino acids.

A homology-based PCR cloning strategy was used to clone DAHP synthase by amplifying a partial DAHP cDNA sequence using the UV-B induced *C. roseus *suspension cell λZAP cDNA library as template and two DAHP gene-specific oligonucleotide primers. The PCR fragment of the expected size was cloned and sequenced. The deduced amino acid sequence of the PCR fragment showed strong homology to known DAHP synthase primary structure. The PCR fragment was used as a probe to screen the *C. roseus *cDNA library (2 × 10^5 ^plaque forming units). This led to the isolation of a full-length cDNA clone.Its insert DNA was completely sequenced. The cDNA contains an open reading frame of 1361 nucleotides and encodes a deduced protein of 446 amino acid residues with a calculated molecular weight of 53 kDa. The amino acid sequence of the DAHP synthase showed high homology with the known nuclear coded chloroplast DAHP synthases of plant origin: *S. tuberosum *(79%) *L. esculentum *(75%) and A*. thaliana *(75% and 77%) (Fig. 1) and revealed the presence of a chloroplast signal peptide at the N-terminus of the sequence (250-273nt).

### Accumulation of CrDHS1 synthase transcript on UV-B irradiation

**Figure 2:**
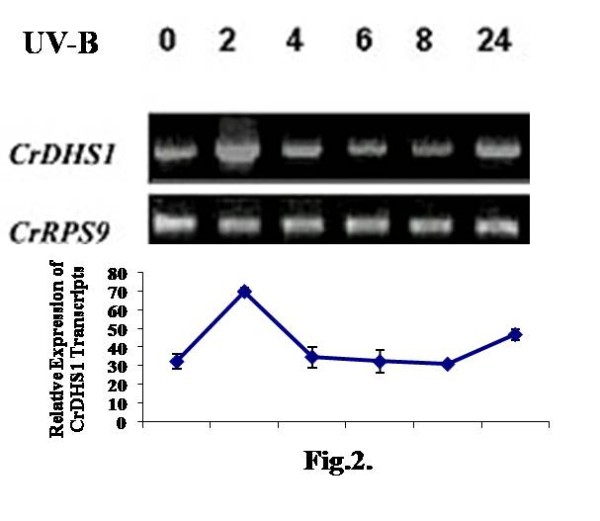
**Expression patterns of *CrDHS1 *in response to UV-B irradiation in *C. roseus *cultured cells**. Six-day-old cultured cells were irradiated for 5 min with UV-B light or left untreated, harvested at the indicated time points, and total RNA was isolated and used for RT-PCR as described in materials and methods. Equal cDNA amounts were ensured by amplification of constitutively expressed Rps9 gene as positive control. Quantification of *CrDHS1 *and *CrRPS9 *expression was performed. The mean ± S.D. values of three independent experiments using Image Guage software (version 2.54) are shown in an inset. Each figure shows visual (gel plots) and graphical representation of the expression

The suspension cultured cells of *C. roseus *were irradiated with a 5 min pulse of UV-B light. Total RNA was extracted from irradiated or untreated cells at various time points up to 24 h and analyzed. *CrDHS1 *synthase transcript accumulation was up regulated under the influence of UV-B irradiation significantly only at 2 h interval relative to the untreated cells which is shown in fig. 2 both in visual and. in graphic representation As anticipated, the *CrRPS9 *transcript encoding the 40 s ribosomal protein S9 was not affected by UV-B irradiation. This indicates specificity of the effect of UV-B treatment in the accumulation of *CrDHS1 *synthase transcripts.

### UV-B-induced expression of CrDHS1 is inhibited by suramin

**Figure 3:**
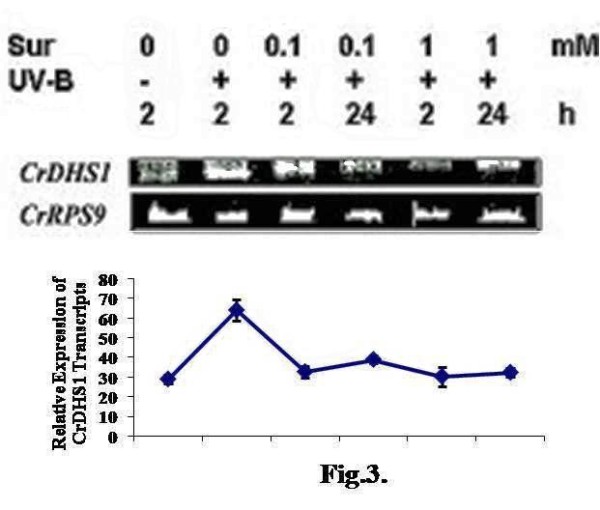
**Effect of suramin on UV-B-induced expression of *CrDHS1***. Six-day-old cultured cells were treated for 10 min with 0.1 or 1 mM suramin, and were then irradiated with UV-B for 5 min. As control, one set of cells were irradiated with UV-B alone or left untreated. Other details are as in the legend to Fig. 2.

Suramin, a polysulfonated compound interferes with the binding of growth factors and cytokines to their respective plasma membrane receptors [7]. Treatment of *C. roseus *cells with 0.1 and 1 mM suramin concentrations resulted in the inhibition of UV-B-induced accumulation of *CrDHS1 *transcript (Fig. 3) and this was not restored even at 24 h indicating the irreversible nature of the effect of suramin treatment under these conditions. These data indicate that suramin-sensitive cell surface receptor participates in the UV-B-induced expression of *CrDHS1 *gene.

### Role of Ca^2+ ^in UV-B-induced regulation of CrDHS1 mRNA levels

**Figure 4:**
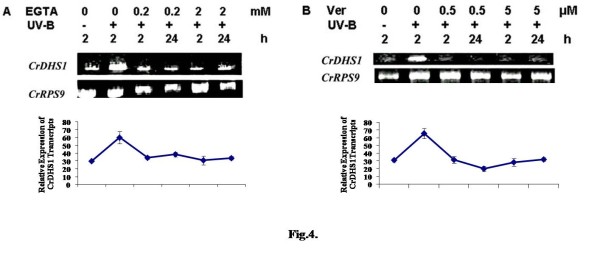
**Effect of EGTA and verapamil on UV-B-induced expression of *CrDHS1***. Six-day-old cultured cells was treated for 10 min with 0.2 or 2 mM of EGTA (*A*) or 0.5 or 5 μM of verapamil (*B*), followed by 5 min of UV-B irradiation. Other details are as in the legend to Fig. 2.

The UV-B induced *CrDHS1 *transcript accumulation was examined in the cells that were pre-treated with a specific calcium chelator, EGTA. Chelation of extracellular Ca^2+ ^with EGTA inhibited the UV-B-induced expression of *CrDHS1 *mRNA (Fig. 4). The inhibition of this accumulation was dose-dependent. Treatment with verapamil, a plasma membrane Ca^2+ ^channel blocker, also inhibited the UV-B-induced *CrDHS1 *transcript accumulation completely at both the concentrations and time periods (2 h and 24 h) checked (Fig. 4). This shows that the UV-B-induced expression of *CrDHS1 *gene is due to the elevation of intracellular Ca^2+ ^concentrations.

### Role of protein kinases and protein phosphatases in UV-B-induced regulation of CrDHS1 mRNA levels

**Figure 5:**
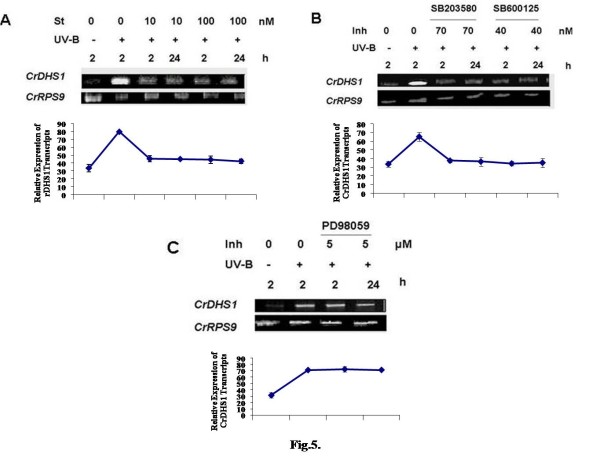
**Effect of protein kinase inhibitors on UV-B-induced expression of *CrDHS1***. Six-day-old cultured cells were treated for 10 min with 10 or 100 nM staurosporine (*A)*, 70 nM SB203580 (P38 inhibitor) (*B*), 40 nM SB600125 (JNK inhibitor) (*B) *or 5 μM PD98059 (ERKK inhibitor) (*C*), followed by 5 min of UV-B irradiation. Other details are as in the legend to Fig. 2.

Specific inhibitors of protein kinases *viz*., staurosporine, a potent inhibitor of serine-threonine kinases; SB 203580, an inhibitor of P38 class of MAP kinase; PD 98059, an inhibitor of ERKK class of MAPKK, and SB 600125, an inhibitor of Janus kinases were used to determine whether protein phosphorylation/dephosphorylation is involved in UV-B-induced *CrDHS1 *transcript accumulation. As shown in Fig. 5, pre-treatment with 10 and 100 nM concentrations of staurosporine completely abolished the UV-B-induced *CrDHS1 *transcript accumulation indicating the involvement of staurosporine-sensitive kinase(s). The addition of SB 203580 and SB 600125 inhibitors to the cell suspension cultures prior to UV-B irradiation also effectively blocked the UV-B-induced *CrDHS1 *transcript accumulation. The effect of each inhibitor treatment was persistent even at 24 h after irradiation. However, pre-treatment with PD 98059 did not inhibit the UV-B-induced *CrDHS1 *transcript accumulation. The results showed that the activation of MAPK cascade is necessary for the UV-B-induced accumulation *CrDHS1 *mRNA as the said compounds are specific inhibitors of MAPK.

**Figure 6:**
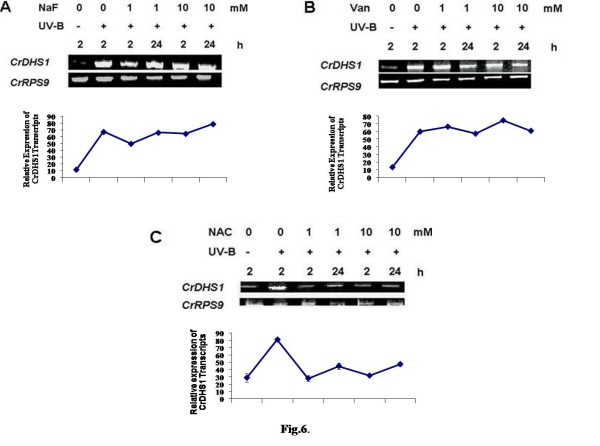
**Effect of NAC, NaF and orthovanadate on UV-B-induced expression of *CrDHS1***. Six-day-old cultured cells were treated for 10 min with 1 and 10 mM of N-acetyl cysteine (*A*), 1 and 10 mM of NaF (*B*), or 1 and 10 mM of orthovanadate (*C*), followed by 5 min of UV-B irradiation. Other details are as in the legend to Fig. 2.

Pre-treatment of cells with protein phosphatase inhibitors showed the opposite effect of kinase inhibitors on the UV-B-induced *CrDHS1 *transcript accumulation (Fig. 6). The UV-B-induced *CrDHS1 *transcript accumulation (Fig. 6B and 6C) was stimulated by the addition of orthovanadate, a known inhibitor of protein-tyrosine phosphatases [8] or sodium fluoride, a strong inhibitor of serine-threonine phosphatases [9]. NAC a known protectant of the thiol group of phosphatases from inactivation [8] had exactly the opposite effect of phosphatase inhibitors. Pretreatment of cells with NAC at 1 and 10 mM concentrations inhibited the accumulation of *CrDHS1 *transcript (Fig. 6A). These results indicate that phosphorylation and/or dephosphorylation events are involved in the UV-B-induced transcription of *CrDHS1 *gene.

## Discussion

We reported in a previous study, exposure of *C. roseus *cultured cells to UV-B irradiation induced the transcription of genes encoding tryptophan decarboxylase and strictosidine synthase and the catharanthine accumulation [10,11]. Tryptophan decarboxylase (TDC) and strictosidine synthase (STR) are key enzymes of terpenoid indole alkaloid biosynthesis and their expressions increases on UV-B irradiation in *C. roseus *plants and cells [12]. DAHP synthase is a key enzyme in the shikimate pathway of the primary metabolism involved in the biosynthesis of tryptophan that serves dual purposes as substrate in synthesis of proteins and of terpenoid indole alkaloids. UV-B is also known to induce the production of phenolics in plants [13-17]. These require the shikimate pathway and phenylalanine and hence DAHP synthase. It is possible that UV-B -induced DAHP synthase plays a role in UV-B induced accumulation of catharanthine and other terpenoid indole alkaloids in *C. roseus *cultured cells.

In the study, the investigation on the signal transduction pathway for *CrDHS1 *mRNA accumulation induced by UV-B irradiation in *C. roseus *cultured cells showed that a suramin-sensitive cell surface receptor is involved in the UV-B mediated signal pathway (Fig. 3).

Treatment with Ca^2+^-chelator (EGTA) and the Ca^2+^-channel blocker (verapamil) blocked the UV-B-induced accumulation of *CrDHS1 *transcript (Fig. 4A and 4B). These results suggest that UV-B-induced accumulation of *CrDHS1 *transcript is mediated by Ca^2+^. EGTA and verapamil are unlikely to enter cells, and verapamil blocks the Ca^2+ ^channels localized in the plasma membrane [18,19]. UV-B irradiation appears to influence the activity of the Ca^2+ ^channels. The data also indicate that the influx of Ca^2+ ^from extracellular space. Ca^2+ ^signaling often co-ordinates parallel and/or sequential use of different sources of Ca^2+^, and different channels in different sub-cellular locations. Thus, the present study provides the evidence that Ca^2+ ^serves as a second messenger in UV-B-signal in the expression of *CrDHS1 *gene. Calcium fluxes are known to be involved in a large number of intracellular signaling processes [20,21]. Yeast elicitor was shown to induce a transient increase in cytosolic calcium in *C. roseus *cells, which in turn was necessary for the induction of tryptophan decarboxylase (TDC) and strictosidine synthase (STR) mRNA expression [10,22,23]

The phosphorylation/dephosphorylation of proteins has been thought to play a key role in the transduction of elicitor signals in plant cells. Experimental findings in this study also demonstrated that protein phosphorylation is involved in UV-B induction of *CrDHS1 *transcript accumulation. Many MAPKs and CDPKs have previously been reported to play essential roles in elicitor-induced production of plant secondary metabolites [24,25]. The observed inhibitory effects of protein kinase inhibitor staurosporine and MAPK cascade inhibitors SB203580 and SB600125 showed that staurosporine-sensitive CDPK and p38-type and c-Jun MAP kinases play important roles in the UV-B-induced *CrDHS1 *transcript accumulation. We also observed that pre-treatment with protein phosphatase inhibitors, orthovandate and sodium fluoride, stimulated the UV-B-induced *CrDHS1 *mRNA levels. The evidence presented in this study indicates the participation of both protein kinase and protein phosphatase activities in control of the UV-B induced *CrDHS1 *expression.

**Figure 7:**
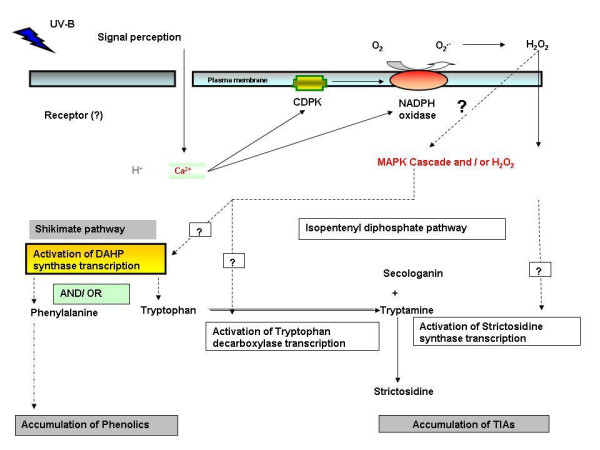
**Model for UV-B-induced transcript accumulation of *CrDHS1***.

Model for UV-B-induced transcript accumulation of *CrDHS1 *has been proposed based on the previous and current findings (Fig. 7).

## Materials and methods

### Chemicals

EGTA, N-acetyl cysteine, sodium fluoride, sodium orthovanadate and verapamil were purchased from Sigma Chemical Company, St. Louis, USA. Staurosporine and suramin were obtained from MP Biomedicals, Germany. SB 203580 (P38 inhibitor), PD 98059 (ERKK inhibitor) and SB 600125 (JNK inhibitor) were gifted by Prof. Anjali Karande, I.I.Sc, Bangalore.

### Cell culture and treatments of cells with UV-B and chemicals

*C. roseus *suspension-cultured cells were cultivated as described previously [26]. In brief, cells were subcultured weekly and stationary phase suspension cultured cells (6-day-old) were used for treatments with UV-B and inhibitors. The cells were treated for 10 min with chemicals and subsequently irradiated with UV-B for 5 min. Control cultures were treated with an equivalent amount of water, ethanol or DMSO. Cells were harvested at indicated time points, immediately frozen in liquid N_2 _and stored at -80°C until used for RNA extraction.

### *Catharanthus roseus *cDNA library construction

A *C. roseus *cDNA library was constructed to facilitate the isolation of cDNA encoding DAHP synthase as well as for other genes. Total RNA was isolated from UV-B-irradiated C*. roseus *suspension cultured cells using Qiazol reagent (Qiagen Inc. Germany) according to the manufacturer's instructions. The Poly(A)^+^RNA was purified from this total RNA by chromatography on oligo (dT)-cellulose (Qiagen) and 5 μg of the resulting mRNA was utilized to construct a cDNA library using a λ ZAP II-cDNA synthesis kit and ZAP-cDNA gigapack III gold packaging kit (Stratagene) following the manufacturer's instructions.

### Preparation of the probe of DAHP synthase and cDNA library screening

Gene specific primers (forward primer **- **5'-ATG GGT GGG GGA ACG TAC GAG ACA -3' and reverse primer -5'-AGG AGA ATG GGC GTT GAG TAC CGA-3') were designed based on the conserved nucleotide region of known DAHP synthases from *A. thaliana *(M74819 and M74820), *L. esculentum *(Z21792) and *S. tuberosum *(M95201), and used to amplify 486-bp fragment in a PCR reaction (35 cycles of 94°C for 1 min, 54°C for 1 min, 72°C for 1 min) using UV-B-induced *C. roseus *library phage cDNA as template. The amplified fragment was cloned in the plasmid vector pMOS Blue and sequenced in an automated DNA sequencer (PRISM™Ready Reaction DyeDeoxy™Terminator Cycle Sequencer). The sequence result revealed that this gene fragment (designated probe DAHPS) was a part of the *C. roseus *DAHP synthase gene. The partial DAHP synthase cDNA was labeled with α^32^P-dCTP using random primer labeling kit (Fermentas) and used to screen the *C. roseus *cDNA library. A plaque showing positive signal was purified through additional two rounds of hybridization. The purified λZAP II cDNA clone was mass excised in vivo as pBluescript SK (-) plasmid in *Escherichia coli *host strain SOLR (Stratagene) and the purified plasmid was sequenced.

### Nucleotide and protein sequence analysis

For comparison and analysis of the sequence data, the following programs were used: BLAST [27], FASTA, GAP, MAP, SEQED and TRANSLATE of Genetics Computer Group (GCG), Wisconsin, version 7.0 [28]. The multiple sequence alignment was performed using the CLUSTALW, European Bioinformatics Institute, at the ExPasy site http://www.expasy.ch[29]. The nucleotide sequence reported in this work has been deposited in the GenBank database under the accession number DQ859024.

### RNA isolation and reverse transcription and polymer chain reaction (RT-PCR) analyis

Total RNA from the suspension cultured cells of *C. roseus *exposed to UV-B and/or pretreated with different chemicals was isolated using the Qiazol reagent (Qiagen Inc. Germany) following the manufacturer's instructions. The RNA samples were quantified by spectrophotometry at wavelengths 260 and 280 nm (A_260_/A_280 _~ 2.0; A_260 _= 40 μg RNA/ml), and visual inspection in agarose gel(s). DNA was removed from total RNA samples by treatment with RNase-free DNase I. Reverse transcription was carried out in a 20 μl reaction mixture containing 1 μg of total RNA, 5 μg oligo d(T)_16-18 _primer, MuMLV reverse transcriptase (40 U), RNasin (20 U), 0.5 mM dNTPs and MuMLV reverse transcriptase reaction buffer (250 mM Tris-HCl, pH 8.3, 250 mM KCl, 20 mM MgCl_2 _and 50 mM DTT) at 37°C for 1 h, and terminated by heating at 70°C for 10 min. After the RT reaction, the cDNA was subjected to PCR reactions. The following pairs of primers were used: 5'-ATGCTGCGGAGCCTGCCAAGACAC-3', as the forward primer and 5'-AGGAGAATGGGCGTTGAGTACCGA-3', as the reverse primer for *C. roseus *DAHP synthase (*CrDHS1*), and 5'-TTAGTCTTGTTCGAGTTCATTTTGTAT-3', as forward primer and 5'-GAGCAAATTAACTCAATTGATAATTAAC-3', as reverse primer for the ribosomal protein 9 gene (*CrRPS9*). A common PCR mix for *CrRPS9 *and *CrDHS1 *for each treatment was prepared containing one μl of the RT reaction mix per 20 μl reaction volume containing 0.4 U of Taq DNA polymerase (Fermentas), 0.1 mM dNTP (Fermentas) and 200 μM of each dNTP and later split into equal half to add 100 pM of gene-specific primer in a 1X reaction buffer. Reactions were amplified for a total of 15 cycles in the Minicycler (MJ Research PTC-150) using 94°C denaturation (1 min), 52°C annealing for *CrDHS1 *and 50°C for *CrRPS9 *(1 min) and 72°C extension (1 min), followed by a further 5 min extension. The RT-PCR products were separated by electrophoresis in 1% agarose gel(s), stained with ethidium bromide, and photographed under UV light using Alpha Imager 2200 (Alpha Innotech Corporation, San Leandro, CA). RT-PCR analysis of *CrRPS9 *was used as a control for testing RNA integrity and accuracy of loading. PCR products of the expected sizes were obtained and their identity confirmed by sequencing in all cases. Quantification of *CrDHS1 *and *CrRPS9 *was performed by densitometry using the Image Guage software (version 2.54). The averages of three independent quantifications of each experiment for *CrDHS1 *were used for plotting the graph, where the error bar represents the standard deviation. The increase observed at 2 h was found to be significant.

## Abbreviations

*CrDHS1: Catharanthus roseus *DAHP synthase (enzyme/gene); DAHP: 3-deoxy-D-arabino-heptulosonate-7-phosphate; ERKK: Extracellular regulated kinase kinase; JNK: Janus kinase; MAPK: Mitogen-activated protein kinase; P38: P38 kinase (MAPK); ROS: Reactive oxygen species; STR: Strictosidine synthase; TIAs; Terpenoid indole alkaloids; TDC: Tryptophan decarboxylase

## Competing interests

The authors declare that they have no competing interests.

## Authors' contributions

CJB provided project leadership and financial support. Experiments were designed by all the three authors and performed by SR and NDP. CJB, SR and NDP wrote the manuscript, which all the authors read and approved.
